# Tuning Porosity of Reduced Graphene Oxide Membrane Materials by Alkali Activation

**DOI:** 10.3390/nano10112093

**Published:** 2020-10-22

**Authors:** Yang Shen, Luca Maurizi, Giuliana Magnacca, Vittorio Boffa, Yuanzheng Yue

**Affiliations:** 1 Department of Materials Science and Engineering, Qilu University of Technology (Shandong Academy of Sciences), Jinan 250353, China; ysh@qlu.edu.cn; 2 Department of Chemistry and Bioscience, Aalborg University, 9220 Aalborg, Denmark; 3 Dipartimento di Chimica, Universitá di Torino, 10125 Torino, Italy; lucam@build.aau.dk (L.M.); giuliana.magnacca@unito.it (G.M.)

**Keywords:** graphene oxide, potassium hydroxide, thermal activation, membrane materials

## Abstract

The alkali-activation method allows for obtaining highly porous carbon materials. In this study, we explored the effect of activation temperature and potassium hydroxide concentration on the pore structure of reduced graphene oxide (rGO), as potential membrane material. Above 700 °C, potassium species react with the carbon plane of rGO to form nanopores. This activation process is deeply studied through DSC measurements and isothermal gravimetric analysis. The porosity of the formed materials consists of both micro- and mesopores, with most of the pores having a size smaller than 10 nm. The specific surface area and pore volume increase with increasing the potassium hydroxide/graphene oxide weight ratio (KOH/GO) up to 7 (897 m^2^∙g^−1^ and 0.97 cm^3^∙g^−1^, respectively). However, for a synthesis mixture with KOH/GO of 10, the specific surface area of the produced material drops to 255 m^2^∙g^−1^. The film-forming ability of the porous reduced graphene oxide (PRGO) was tested by drop-casting on porous silicon carbide substrates. In this case, continuous PRGO films were obtained only from dispersions with 5 g∙L^−1^ GO loading and KOH/GO ≤3. Such films can still have high specific surface area and pore volume (up to 528 m^2^∙g^−1^ and 0.53 cm^3^∙g^−1^) and main pore volume <10 nm. Hence, they can potentially be applied as membrane devices, but their scalability and their adhesion on the substrate under realistic filtration conditions still remain challenges.

## 1. Introduction

Because of the unique single-atom-thick structure [1], graphene-based materials have been indicated as material for water purification membranes with outstanding permeability and selectivity by various theoretical studies [2,3,4,5,6,7,8,9,10]. Nevertheless, graphene’s perfect honeycomb aromatic lattice cannot be permeated by any molecule, including water. For this reason, different methods have been proposed to create pores on graphene. For instance, nanopores were created in 20 µm^2^ large graphene sheets by oxygen plasma etching, which also allowed for tuning pore size. Such type of membranes combined sodium chloride rejection and an exceptionally high water permeability [11]. However, fabrication of such membranes over large areas remains a challenge, thus preventing their practical application. On the contrary, graphene oxide (GO) can be easily prepared and processed. Therefore, GO is attracting enormous attention for water purification membranes [2,12,13]. GO is an oxidized form of graphene, which contains various types of functional groups as hydroxyl, carbonyl, carboxyl, and epoxide [14]. Due to the presence of these oxygen groups, GO can be easily dispersible in water and therefore simply processed [15,16]. Indeed, GO is certainly the most easily scalable precursor of graphene. Moreover, the oxygen functional groups expand the interlayer distance of the carbon layers from 0.34 nm in graphite to 0.7–0.8 nm in GO [17]. Such expended interlayer spacing allows water molecules to permeate GO films [18,19], making them suitable for the fabrication of membranes for water desalination and detoxification. However, neat GO membranes are not thermally and chemically stable and they have the tendency to exfoliate in aqueous solution [20]. On the contrary, reduced graphene oxide (rGO) membranes possess high thermal and chemical stability [21,22], but their structure resembles the dense packing of laminas of graphite, thus preventing water permeation. In this context, how to apply the remarkable rGO as membrane material deserves to be explored.

Recently, porous reduced graphene oxide (PRGO) has been fabricated by activation with potassium hydroxide (KOH) [23,24,25]. This method is similar to one of the most common procedures for the synthesis of activated carbons. Impregnation with aqueous KOH and heating at high temperature produce nanopores in the multilayered GO films. Above a certain pore density, an interconnected channel network is formatted across the membrane materials, thus allowing for permeation. However, high KOH loadings prevent the formation of a continuous membrane layer and/or cause its detachment from the membrane support. Here, we studied the optimal conditions to obtain highly porous PRGO, without compromising the integrity of the material. Differential scanning calorimetry-differential thermogravimetric analysis (DSC-TGA) measurements were carried out to understand the kinetics and chemical mechanisms of pore formation. Low-temperature nitrogen adsorption porosimetry and electron microscopies were performed in order to characterize the PRGO pore structure.

## 2. Experimental

### 2.1. PRGO Synthesis

All the chemicals used for the synthesis of the nanocomposites were purchased from Sigma–Aldrich (Darmstadt, Germany), unless otherwise specified. Graphene oxide was synthesized via Hummers’ method: 2.0 g graphite (Graphit Kropfmühl GmbH, Hauzenberg, Germany) was mixed with 1.0 g NaNO_3_ (99%), 92 mL H_2_SO_4_ (98%), and 12.0 g K_2_MnO_4_ (99%) in a 1 L beaker [26,27]. At first, the mixture was placed in an ice bath and stirred for 30 min. Then, the mixture was moved to a water bath and kept at 35 °C for 1 h, a thick dark green paste could be obtained; later, 100 mL deionized water was slowly poured into the beaker and the reaction temperature was raised to 95 °C, a dark brown suspension could be observed. After adding 500 mL of deionized water, followed by 6 mL of 30% H_2_O_2_ solution, the mixture’s color changed into light yellow. The yellow graphene oxide suspension was washed with 200 mL HCl (5%) one time, and with 500 mL deionized water 5 times to remove impurities’ ions. The so obtained graphene oxide (GO) slurry was freeze-dried and kept in this form until it was used for PRGO synthesis. The obtained cleaned graphene oxide powders were dispersed in deionized water by 30 min stirring and ultra-sonicated for 1 h to exfoliate the GO. Then, different amount of KOH pellets (86%) were added to the dispersion, to achieve GO:KOH mass ratios of 1:0.5, 1:1, 1:3, 1:5, 1:7 and 1:10. After stirring for 12 h to homogenize the distribution of GO flakes and potassium ions, these dispersions were ultra-sonicated in water for another 1 h.

### 2.2. PRGO Characterization

An aliquot of the GO/KOH dispersions was dried at 40 °C in air for 48 h. The obtained dry mixtures were used for material analysis.

Differential scanning calorimetry (DSC) and Thermogravimetry (TG) measurements were performed on a Simultaneous Thermal Analyzer 449C Jupiter (Netzsch, Selb, Germany). The mixed samples (ca. 4.5 mg) were placed into a platinum crucible at room temperature. In the dynamic measurements, the samples were held for 5 min at an initial temperature of 40 °C and then heated to 1000 °C at a rate of 10 °C min^−1^ in an argon atmosphere. These mixed samples were also tested in Isothermal gravimetric measurements in argon atmosphere with around 10 mg samples, then were heated to the target temperature 600, 620, 640, 660, 680, and 700 °C at 40 °C∙min^−1^ and held for 5 h.

Some of mixed samples were annealed at 700 °C at a rate of 10 °C min^−1^ for 1 h in Ar atmosphere to produce PRGO samples. N_2_ adsorption measurements were performed at 77K on a gas-volumetric apparatus ASAP2020 (Micromeritics, Norcross, GA, USA). Around 25 mg samples were outgassed at 250 °C for about 4 h under vacuum (residual pressure 10^−2^ mbar) to degas the water and impurities before the measurement. Specific surface areas were determined using the Brunauer–Emmett–Teller (BET) model and porosity was studied applying the Density Functional Theory (DFT) method using slit pores shape and small regularization [28,29]. X-ray diffraction patterns (XRD) were obtained on a XRA 888/D (PANalytical, Almelo, The Netherlands) with Cu Kα radiation in the 20 range between 5° and 80°. Scanning electron microscope (SEM) images were taken at an accelerating voltage of 10 kV. High-resolution transmission electron microscopy (HR-TEM) images were taken on a JEOL 3010-UHR (acceleration potential: 300 kV). Samples for TEM investigation were supported onto holed carbon coated copper grid by dry deposition.

### 2.3. Film Deposition

Flat sheet 1 × 1 cm^2^ SiC substrates (nominal pore size = 0.04 µm, LiqTech International A/S, Ballerup, Denmark [30]) were dipped in different GO:KOH dispersions for 30 s and then dried for 24 h at room temperature. Three samples were prepared from each dispersion. The membrane samples were annealed at 700 °C (heating rate of 1 °C∙min^−1^, dwell time of 1 h) in Ar atmosphere to produce porous GO membranes.

## 3. Results and Discussion

### 3.1. Thermal Activation

**Figure 1:**
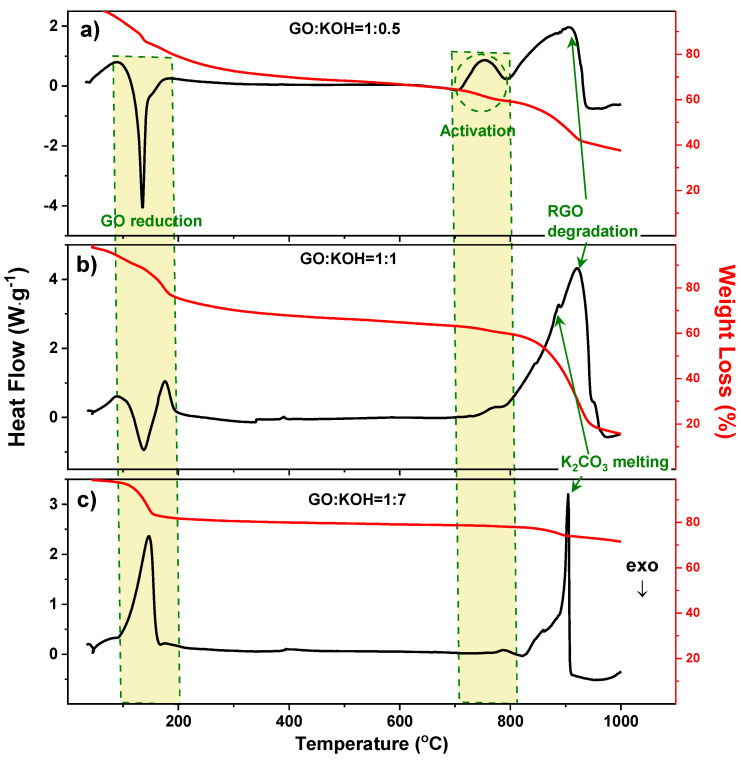
DSC (black curves) and TG (red curves) analysis of samples with GO:KOH = (**a**) 1:0.5; (**b**) 1:1; (**c**) 1:7. Yellow areas indicate the temperature ranges at which GO reduction and alkali activation occur.

Simultaneous Differential Scanning Calorimetry (DSC)-Thermogravimetry (TG) was used to investigate the thermal evolution of the GO-KOH samples. The DSC curves of three samples prepared with a KOH/GO weight ratio of 0.5, 1, and 7 are shown in Figure 1. The samples with KOH/GO of 0.5 and 1 show the typical DSC profiles of GO material, i.e., they show a clear exothermal peak between 100 and 210 °C [31], which arises from the reduction of GO, which consists in the degradation of the most liable functional groups on the carbon network, as confirmed by the weight loss in the TG curve. However, the area of this exothermal peak largely decreases with increasing the KOH/GO ratio from 0.5 to 1. In the case of the sample with KOH/GO = 7, the exothermal peak has totally disappeared and only a weak endothermic response left (Figure 1c). This trend is not surprising because it has been reported that, in strong bases, it can induce partial degradation of GO functional groups even at room temperature [32,33], Therefore, GO samples with high KOH content were partially reduced before DSC analysis. In addition, KOH crystals dispersed in GO can easily adsorb moisture from the ambient. The adsorbed water would evaporate above 100 °C, yielding the endothermic peak (DSC curve) and the mass loss (TG curve) in Figure 1c.

Above 700 °C, all the samples present endothermic responses. The curve of the sample with KOH/GO = 0.5 shows two endothermic peaks between 700 and 950 °C. According to the previous studies on the fabrication of active carbons from anthracites [34,35,36], degradation of the carbon framework occurs in this temperature range, involving the following reactions:

(1) $$6\mathrm{KOH}+C⇄2K+3H_{2}+2K_{2}{\mathrm{CO}}_{3}$$

(2) $$K_{2}{\mathrm{CO}}_{3}+C\;⇆K_{2}O+2\mathrm{CO}$$

(3) $$K_{2}{\mathrm{CO}}_{3}\;⇆{\;K}_{2}O+{\mathrm{CO}}_{2}$$

(4) $$2K+{\mathrm{CO}}_{2}\;⇆K_{2}O+\mathrm{CO}$$

Above 700 °C, the potassium ions would react with the carbon atoms in six carbon rings, resulting in the production of carbonates and the formation of pores on the graphene sheet. The melting point of the by-products K_2_CO_3_ is around 891 °C [37,38]. Above this temperature, the increased interface between the melted K_2_CO_3_ and the rGO can facilitate further degradation of the carbon-based material. Therefore, the second endothermic peak may correspond to combination of the melting of K_2_CO_3_ and the chemical degradation of rGO, which is confirmed by the mass loss in the TG curves. The peak centered at about 890 °C is dominant in samples with high KOH/GO weight ratio. Here, we infer that large quantities of K_2_CO_3_ are produced so that the melting DSC response covers the other signals.

**Figure 2:**
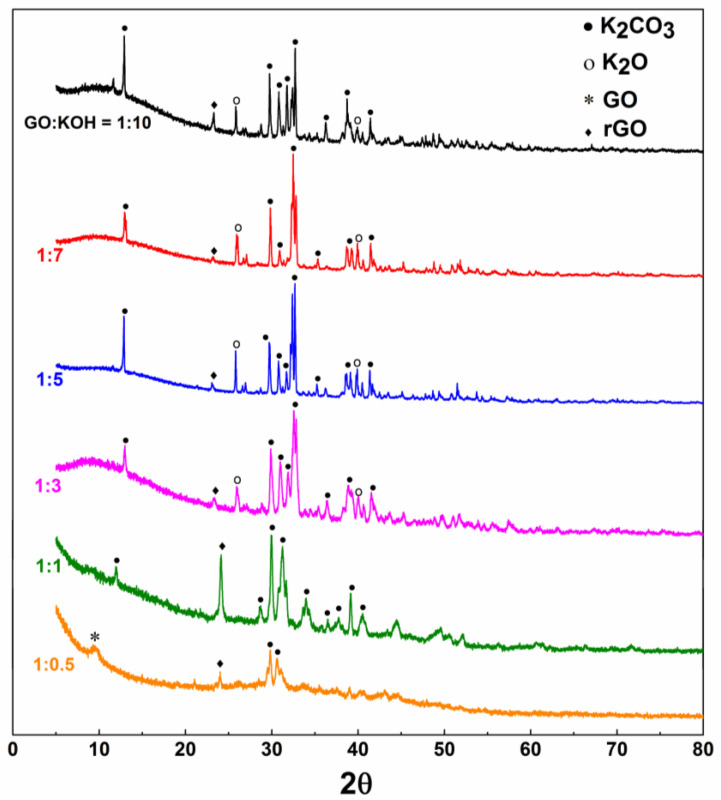
X-ray diffraction patterns of GO-KOH samples with KOH/GO weight ratio = 0.5, 1, 3, 5, 7 and 10 after annealed at 700 °C for 1 h.

Figure 2 shows the X-ray diffraction patterns of samples with six different KOH/GO mass ratios, namely 0.5, 1, 3, 5, 7 and 10, after been annealed at 700 °C for 1 h. All the XRD curves present the typical rGO peak at around 25° [39]. The other peaks can be assigned to K_2_CO_3_ and K_2_O (Jade PDF card 49-1093 and 26-1327, respectively) [34,35], supporting the above reported reactions (Equations (1)–(4)).

**Figure 3:**
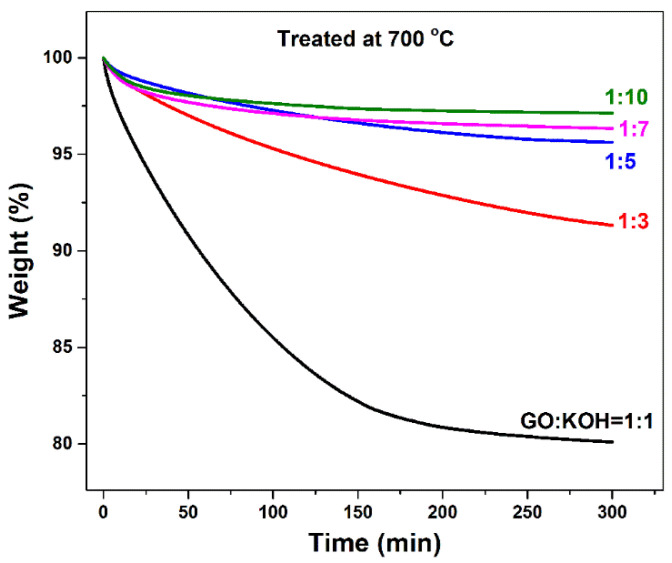
Isothermal TG curves of five different KOH/GO mass ratio samples annealed at 700 °C for 5 h.

Figure 3 shows the Isothermal Gravimetric Analysis of the five samples’ annealing process for 5 h at 700 °C. The 1:1 sample presents largest mass loss, i.e., nearly 20%. Besides the evaporation of physical adsorbed water and the decomposition of functional groups [31], the graphene carbon plane would generate a porous structure after being activated by KOH at high temperature. With increasing the amount of KOH, the mass loss decreases gradually. Around a 10% mass loss could be observed for the 1:3 sample. When KOH/GO reaches 10, less than 3% mass loss is detected. Normally, when a larger quantity of KOH is added, more pores would be generated in GO carbon layers, resulting in a bigger mass loss. However, Figure 3 profiles show an inverse trend that more content of KOH leads to less mass loss. We infer that, because of the electronegativity of the surface of GO layers [40], the addition of KOH could break the charge balance and massive functional oxygen groups in GO would already be decomposed so that quite a small proportion of weight loss could be detected during the TG measurement.

**Figure 4:**
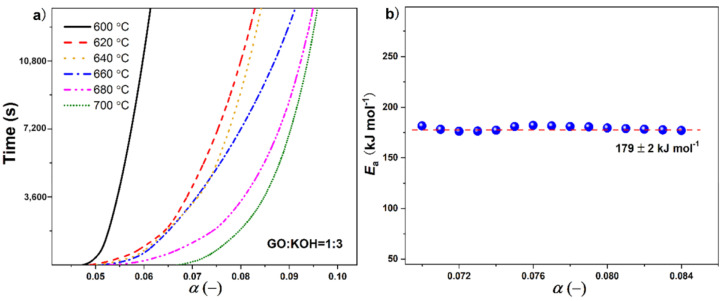
Isothermal Gravimetric Analysis of GO:KOH = 1:3 sample. (**a**) the isothermal TG curves at different temperatures; (**b**) the calculated activation energy. The horizontal dashed line is used for the visual.

Following these observations, the mechanism of activation process is investigated by Isothermal Gravimetric Analysis. as shown in Figure 4. The GO:KOH = 1:3 sample is picked to be measured at six different temperatures for 4 h in Argon atmosphere: 600, 620, 640, 660, 680, and 700 °C. The degree of activation is defined as the fractional mass loss (*α*) of the sample. Figure 4a shows the time, *t*, as a function of *α* for six profiles. As expected, the isotherms in six curves have different slops, since higher temperature treatment results in more pore formation. At 600 °C, the relative mass loss is around 1.44%. This *α* value is kind of small compared to the mass loss during low temperature GO reduction process because carbon plane is very stable in inert gases below 1000 °C, leading to the activation process not being a strong chemical reaction. With the increase of temperature, the fractional mass loss increases to 4.2% at 700 °C. Higher temperature assists the KOH treated GO with generating more defects on a graphene plane.

The apparent activation energy, *E_a_*, for which such process is calculated from the isotherms by using the MacCallum method [41,42], according to the degradation time (*t*), can be expressed as follows:

(5) t=f(α)·A·exp(EaRT)

where *f*(*α*) is an undefined function of the GO+KOH activation, *A* is the pre-exponential factor, and *R* is the universal gas constant. The resulting *E_a_* vs. *α* curve is shown in Figure 4b. Through the whole activation process, the value of *E_a_* is nearly constant, *E_a_* = 179 ± 2 kJ∙mol^−1^, which indicates that the inherent energy barrier for the decomposition of basal carbon plane in GO by KOH activation is constant.

### 3.2. PRGO Morphology

**Figure 5:**
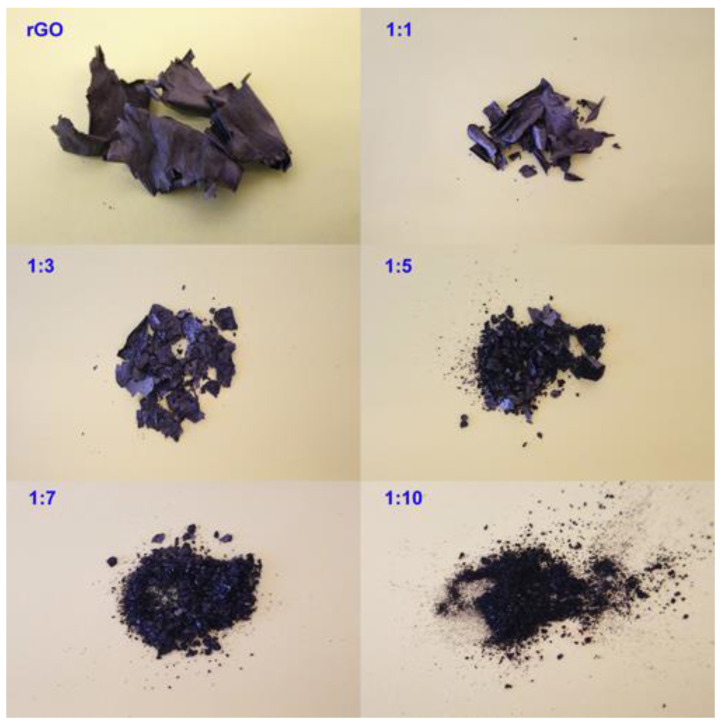
Images of PRGO samples with a different GO:KOH mass ratio without any physical treatment (annealed at 700 °C for 1 h and washed by 5% HCl solution).

Pictures of PRGO samples after thermal treatment at 700 °C for 1 h followed by washing with 5% HCl solution without any physical treatment, like grinding or crushing, are shown in Figure 5. The rGO sample obtained with no addition of KOH keeps its flaky shape after annealing. On the contrary, the addition of KOH causes partial degradation of the carbon materials during heat-treatment and therefore rGO flakes lose the graphene layers’ integrity and break into small fragments. With the increase of KOH content, the consistency of PRGO samples becomes more powdery. The proper ratio of KOH could generate a large quantity of nanopores inside GO; however, an excessive KOH content would have a negative effect on the PRGO integrity.

**Figure 6:**
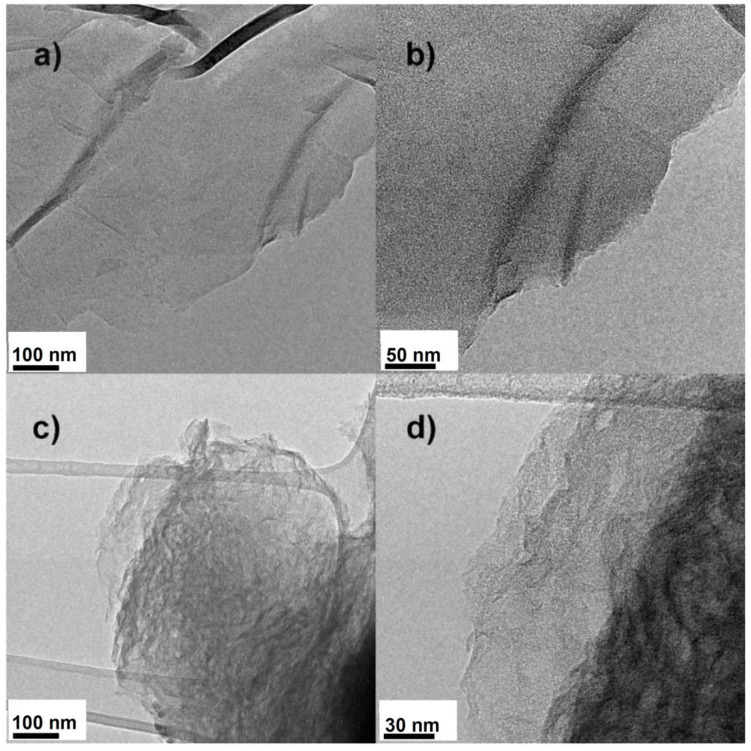
TEM images of the PRGO samples with KOH/GO weight ratio of 0 (**a**,**b**) and 5 (**c**,**d**) after annealed at 700 °C for 1 h.

In order to gain insight into the structure of PRGO carbon network, TEM measurements were carried out as shown in Figure 6. Two samples with KOH/GO = 0 and 5 are selected here to display the change of morphology due to the presence of potassium hydroxide. Figure 6a,b shows the micrograph of the reference rGO sample (KOH/GO = 0). The carbon layers could be exfoliated easily, so that a micrograph of single complete and smooth carbon sheet was obtained. After adding KOH (Figure 6c,d), the carbon sheets become twining and folded, and are more difficult to exfoliate by sonication during the preparation of the sample before analysis. Moreover, the sample activated with KOH presents a high density of disordered and interconnected nanochannels, making it a potential material for molecular separations systems.

**Figure 7:**
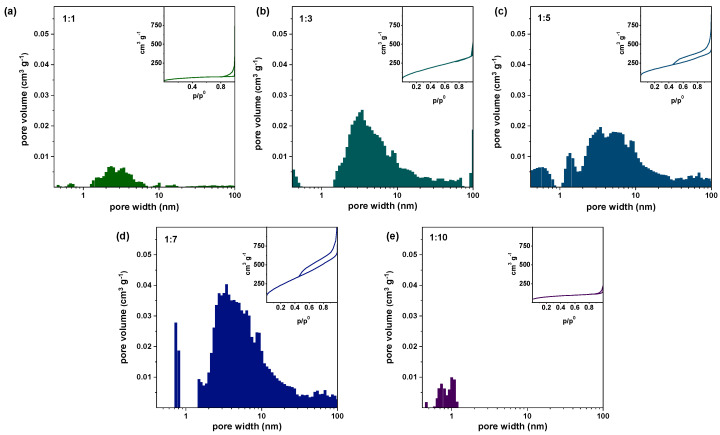
N_2_ adsorption data for PRGO samples with KOH/GO weight ratio (**a**) 1, (**b**) 3, (**c**) 5, (**d**) 7, (**e**) 10 (after annealed at 700 °C for 1 h).

**Figure 8:**
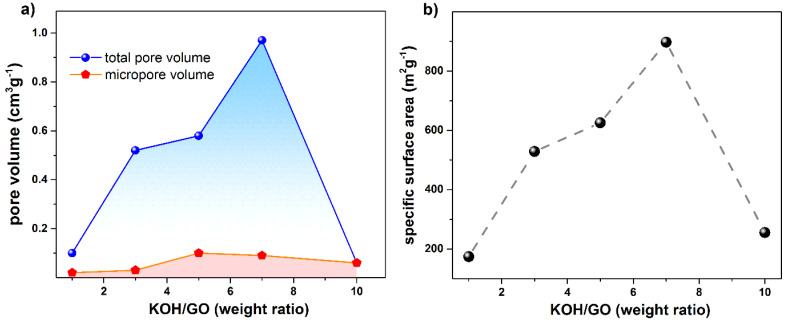
(**a**) DFT specific pore volume and (**b**) BET specific surface area as a function of the KOH/GO weight ratio for the PRGO samples (annealed at 700 °C for 1 h), the dashed line is used for the visual.

The porous structure of the PRGO was analyzed by N_2_ adsorption measurements, as shown in Figure 7. According to IUPAC [43,44], the adsorption curves of all samples type II isotherm, which correspond to monolayer-multilayer adsorption affected by strong surface interaction in defects (pores or wrinkles). All the samples have a broad pore size distribution including micropores and mesopores; meanwhile, the biggest portion of the pore size is located between 0–10 nm. Additionally, pore size distributions are calculated from the adsorption isotherms by using the Density Functional Theory (DFT) method [29], and the obtained results are shown in Figure 8.

The PRGO sample with KOH/GO = 1 has a specific pore volume 0.10 cm^3^∙g^−1^ (in Figure 8a). With the increase in the amount of KOH, the specific pore volume increases as well. In the samples with KOH/GO = 3, 5, and 7, the specific pore volumes are 0.52, 0.58, and 0.97 cm^3^∙g^−1^, respectively. In general, the addition of KOH increases RGO porosity. However, when an excessive amount of KOH is added, the carbon network collapse and the specific pore volume decrease dramatically: it reduces to 0.06 cm^3^∙g^−1^ in the KOH/GO = 10 sample. Consequently, the calculated BET specific surface area presents a similar trend in Figure 8b. The specific surface area of the KOH/GO = 1 sample is 174 m^2^/g. After increasing the amount of KOH, the specific surface area increases to 897 m^2^∙g^−1^ for KOH/GO = 7 sample. However, the surface area decreases with further addition of KOH.

We attribute this trend to the ability of KOH creating defects on the GO basal carbon planes. A moderate amount of KOH would contribute the formation nanopores on the GO graphene sheets. However, when an excessive amount of KOH is added in GO, the carbon network is seriously damaged and the related graphene layers break into small pieces, so that the BET area and the specific pore volume decrease and PRGO samples become more powdery.

As the TEM and N_2_ adsorption measurements show, the PRGO materials present disorder and interconnected porosity, which make them attractive for membrane application. Therefore, we test their film-forming ability, which is another important property for membrane materials.

### 3.3. Film-Forming Ability Tests

**Figure 9:**
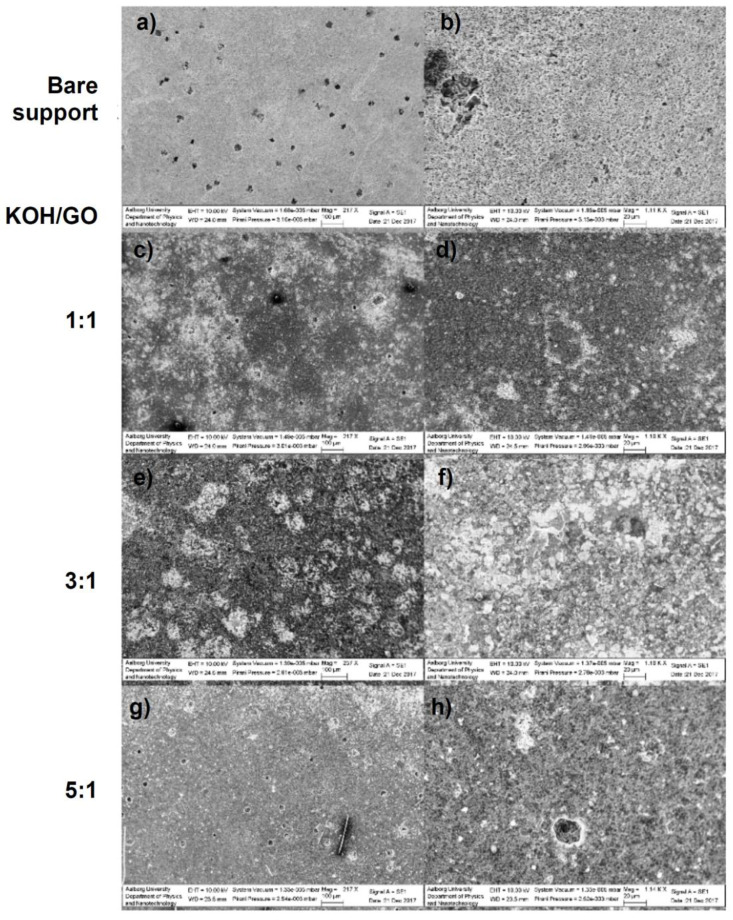
SEM images of the bare silicon carbide flat-sheet support (**a**,**b**) and of the PRGO membranes coated from dispersion with KOH/GO weight ratio of 1 (**c**,**d**), 3 (**e**,**f**), and 5 (**g**,**h**).

Dispersions of GO in aqueous KOH were drop-casted on commercial silicon carbide microfiltration membranes, which are suitable as a substrate for our membrane materials, also in view of the future upscaling of this technology. Membrane fabrication was attempted with different GO loadings in the coating solutions and for different GO/KOH weight ratios. With a GO loading of 3 g L^−1^ in the coating solution, no continuous films are formed. On the other hand, GO loading of 10 g L^−1^ resulted in considerable shrinkage and delamination of the deposited surface from the substrate. Figure 9 reports SEM micrographs of the surface of membranes coated with GO-KOH dispersions (GO loading 5 g L^−1^) after heat-treatment at 700 °C for 1 h in argon atmosphere. The bare support shows an open porous structure with 0.04 µm large pores, but also surface inhomogeneities with size larger than 20 µm. As shown by Figure 9c–f, dispersions with a KOH/GO ratio equal to 1 and 3 yielded continuous layers. However, the PRGO film was not able to coat the largest holes (e.g., defects) on the support surface. On the contrary, dispersion with KOH/GO ratio equal or higher than 5 did not yield a continuous film and most of the material detached from the substrate surface, as shown by the micrographs in Figure 9g,h. The membranes prepared with KOH/GO equal to 1 and 3 show also residual K_2_CO_3_ and K_2_O crystals. With the increase of the KOH amount, these deposits aggregate and form larger crystals. Our attempts to remove these deposits in a stirred beaker containing demineralized water results in damages for the PRGO layers, causing concerns about the mechanical stability of the new membranes under realistic cross-flow conditions. In summary, we were able to synthetize PRGO layers from dispersion of GO in water (5 g L^−1^) and KHO/GO equal to 1 and 3, but their adhesion to the membrane substrate needs to be improved.

## 4. Conclusions

In this work, we explored the thermal evolution of KOH activated porous reduced graphene oxide (PRGO) materials and the influence of KOH loading on their pore structure. Consistently with the high temperature activation process of alkaline treated carbon materials, we generate nanopores in the reduced graphene oxide (RGO) carbon network. Different KOH loading was used (GO:KOH = 1:0.5, 1:1, 1:3, 1:5, 1:7 and 1:10). The calorimetric measurements show that the activation process only occurs above 700 °C. Potassium hydroxide reacts with GO to form K_2_CO_3_, although K_2_O crystals were also detected by XRD. The morphology derived from N_2_ adsorption and TEM measurements has been used to investigate the porous structure after being heat-treated. With the proper amount of KOH, PRGO would have a large specific surface area (maximum 897 m^2^g^−1^). However, when the KOH/GO ratio exceeded 7, the carbon network of the rGO sheets was severely damaged at the detriment of the material integrity and the specific surface area. The film prepared from the dispersion with KOH/GO ratio equal to 7 was not continuous after annealing at 700 °C. On the contrary, continuous layers were formed by dispersions with KOH/GO ratio of 1 and 3, which still were able to generate highly porous materials (Figure 7 and Figure 8). This work indicates that such PRGO materials have the potential for filtration application, while their integrity and their adhesion to the membrane support need to be strengthened (e.g., by pre-treating the SiC porous support with polycarbosilanes, whose conversion to SiC occurs nearly at the same temperature of the alkali activation), and this will be the objective of further study.
